# Blocking *Plasmodium falciparum* Development via Dual Inhibition of Hemoglobin Degradation and the Ubiquitin Proteasome System by MG132

**DOI:** 10.1371/journal.pone.0073530

**Published:** 2013-09-02

**Authors:** Rajesh Prasad, Atul, Venkata Karunakar Kolla, Jennifer Legac, Neha Singhal, Rahul Navale, Philip J. Rosenthal, Puran Singh Sijwali

**Affiliations:** 1 CSIR-Centre for Cellular and Molecular Biology, Hyderabad, Andhra Pradesh, India; 2 Department of Medicine, San Francisco General Hospital, University of California San Francisco, San Francisco, California, United States of America; Johns Hopkins Bloomberg School of Public Health, United States of America

## Abstract

Among key potential drug target proteolytic systems in the malaria parasite *Plasmodium falciparum* are falcipains, a family of hemoglobin-degrading cysteine proteases, and the ubiquitin proteasomal system (UPS), which has fundamental importance in cellular protein turnover. Inhibition of falcipains blocks parasite development, primarily due to inhibition of hemoglobin degradation that serves as a source of amino acids for parasite growth. Falcipains prefer P2 leucine in substrates and peptides, and their peptidyl inhibitors with leucine at the P2 position show potent antimalarial activity. The peptidyl inhibitor MG132 (Z-Leu-Leu-Leu-CHO) is a widely used proteasome inhibitor, which also has P2 leucine, and has also been shown to inhibit parasite development. However, the antimalarial targets of MG132 are unclear. We investigated whether MG132 blocks malaria parasite development by inhibiting hemoglobin degradation and/or by targeting the UPS. *P. falciparum* was cultured with inhibitors of the UPS (MG132, epoxomicin, and lactacystin) or falcipains (E64), and parasites were assessed for morphologies, extent of hemoglobin degradation, and accumulation of ubiquitinated proteins. MG132, like E64 and unlike epoxomicin or lactacystin, blocked parasite development, with enlargement of the food vacuole and accumulation of undegraded hemoglobin, indicating inhibition of hemoglobin degradation by MG132, most likely due to inhibition of hemoglobin-degrading falcipain cysteine proteases. Parasites cultured with epoxomicin or MG132 accumulated ubiquitinated proteins to a significantly greater extent than untreated or E64-treated parasites, indicating that MG132 inhibits the parasite UPS as well. Consistent with these findings, MG132 inhibited both cysteine protease and UPS activities present in soluble parasite extracts, and it strongly inhibited recombinant falcipains. MG132 was highly selective for inhibition of *P. falciparum* (IC_50_ 0.0476 µM) compared to human peripheral blood mononuclear cells (IC_50_ 10.8 µM). Thus, MG132 inhibits two distinct proteolytic systems in *P. falciparum*, and it may serve as a lead molecule for development of dual-target inhibitors of malaria parasites.

## Introduction

Lysosomes and the ubiquitin proteasome system (UPS) are major degradation machineries for damaged and unwanted cellular contents in eukaryotic cells. The UPS degrades damaged and unwanted proteins that are tagged with ubiquitin, whereas large protein aggregates and damaged or dispensable organelles are degraded in the lysosome. Besides housekeeping functions, regulatory roles of these two machineries in development and differentiation are emerging in a number of organisms. Malaria parasites critically rely on these two systems, as inhibitors of the UPS and of proteases of the food vacuole, the lysosome-equivalent of *Plasmodium* , have been shown to block parasite development. Hence, these two proteolytic machineries are attractive targets for antimalarial drug development [1–6].

Erythrocytic malaria parasites critically rely on hemoglobin for amino acids, which is hydrolyzed in the food vacuole by concerted action of several different types of proteases. The major food vacuole-resident hemoglobin degrading proteases are papain-like cysteine proteases falcipains, aspartic proteases plasmepsins, the metalloprotease falcilysin, dipeptidyl aminopeptidase I, and a M1-family alanyl aminopeptidase [7–13]. A large body of literature indicates that the papain-like cysteine proteases falcipains, particularly falcipain-2 (FP2) and falcipain-3 (FP3), are the major hemoglobin degrading enzymes in *P. falciparum* [5]. FP2, FP3 and their homologs in other malaria parasites prefer leucine at the P2 position in substrates and inhibitors [14–24]. Several peptidyl inhibitors of papain-like cysteine proteases, including leupeptin (acetyl-Leu–Leu–Arg-aldehyde, with P2 leucine and aldehyde warhead), block development of malaria parasites by inhibiting FP2 and FP3 [25]. This selectivity for a P2 leucine residue has been exploited for optimizing inhibitors of falcipain-like proteases of malaria parasites [26–28].

Malaria parasites appear to have a typical 26S proteasome [29]. A 26S proteasome is composed of two multi-subunit assemblies: a core protease complex, called the core particle (CP) or 20S proteasome; and a regulatory element, known as the 19S regulatory particle (RP) [30]. The CP is a barrel-shaped complex of four stacked rings, each with 7 α or 7 β subunits, which are arranged as “αββα”. Three catalytically distinct β subunits (β1, β2, and β5) in each inner ring, whose active sites line the central lumen of the CP, form a proteolytic chamber where substrates are degraded. Substrates gain access to this chamber through the pores formed by α rings on either side of the CP [31,32], which requires the RP for opening the pores and unfolding of the substrate. Active site mutagenesis of the three protease subunits together with the use of defined peptide substrates revealed that a typical 26S proteasome possesses three types of activities [33–35]: 1) caspase-like or peptidyl-glutamyl peptide hydrolyzing (PGPH) activity, which cleaves after acidic residues; 2) trypsin-like activity that cleaves after basic residues; and 3) chymotrypsin-like activity, which cleaves after large hydrophobic residues. The majority of available proteasome inhibitors, including MG132, epoxomicin, and lactacystin, block the chymotrypsin activity [6,36–39].

Dual inhibition of falcipains and the UPS with optimal selectivity could offer higher potency and less risk of development of drug resistance by the parasite than individual inhibitors of these two proteolytic systems. One such compound might be MG132 (Z-Leu-Leu-Leu-CHO), as it contains P2 leucine, a falcipain preferred residue, and an aldehyde group that reacts with catalytic cysteine residue of cysteine proteases and threonine residues of protease units of the proteasome. MG132 is a first choice inhibitor for studying UPS in a variety of organisms, including malaria parasites. It is commonly used at micromolar concentrations to study the UPS in a variety of human cell lines, with reported 50% cytotoxic concentrations in the range of 2.5-21 µM depending on the cell type and treatment duration [40–43]. MG132 has also been shown to inhibit the papain-like cysteine proteases cathepsin L and B and the calcium dependent cysteine proteases calpains [44,45].

However, the cysteine protease inhibitory property of MG132 remains underappreciated compared to its extensive use as an UPS inhibitor. Hence, we hypothesized that this compound is a dual-target inhibitor of malaria parasites, blocking hemoglobin degradation by inhibiting falcipains and also inhibiting UPS. To test this hypothesis, we assessed the effects of specific inhibitors of cysteine proteases (E64) and UPS (epoxomicin, lactacystin, and MG132) on parasite development, hemoglobin degradation, proteasome and cysteine protease activities in *P. falciparum* extracts, and activity of recombinant falcipains. We demonstrated that MG132 blocks asexual erythrocytic development of *P. falciparum* by inhibiting both hemoglobin degradation and the UPS.

## Materials and Methods

### Materials

The *P. falciparum* 3D7 strain was obtained from the Malaria Research and Reference Reagent Resource Centre (MR4). MG132, epoxomicin, and lactacystin were from Santa Cruz Biotechnology; all other biochemical reagents were from Sigma or Serva. Plasmid isolation kits were from Qiagen or MACHEREY-NAGEL; cell culture reagents were from Lonza and Invitrogen; restriction and DNA modifying enzymes were from New England Biolabs; and SYBR Green 1 was from Invitrogen. Human blood was collected from healthy volunteers after written consent under medical supervision at the medical dispensary of the institute, and the protocol (IEC-2/2010) for blood collection for this study has been approved by the Institutional Ethical Committee of CCMB.

### Parasite culture

In vitro parasite culture was done according to the protocols approved by the Institutional Biosafety Committees of CCMB and UCSF. *P. falciparum* was cultured in human erythrocytes at 2% haematocrit in the presence of a gas mixture (5% CO_2_, 5% O_2_, and 90% N_2_) in RPMI 1640 medium supplemented with 41.1 mg/litre hypoxanthine, 300 mg/litre glutamine, 2.5% human serum, and 0.5% Albumax II [46]. Synchrony was maintained by serial treatment with 5% D-sorbitol [47]. For parasite isolation, the culture was centrifuged at 894g for 5 min, the supernatant was aspirated off, the pellet was treated with ice-cold 0.05% saponin (in PBS) for 5 min to lyse erythrocytes, and the sample was centrifuged at 12,096g for 5 min at 4°C. The supernatant was discarded, the pellet was washed twice with ice-cold PBS to remove residual hemoglobin, and parasites were recovered by centrifugation at 12,096g for 5 min at 4°C. Genomic DNA (gDNA) was isolated from late trophozoite/schizont stage parasites using the Puregene Blood Core Kit B (Qiagen).

### Parasite growth inhibition assays

Inhibitors of the proteasome (epoxomicin, lactacystin, and MG132), cysteine proteases (L-trans-epoxysuccinyl-leucyl-amido(4-guanidino)butane; E64), and aspartic proteases (pepstatin) were assessed alone and in combinations for inhibition of *P. falciparum* erythrocytic stage development. 200 x stocks of inhibitors were made in DMSO and serially diluted 2-fold in 100 µl culture medium across rows of a 96 well tissue culture plate. Control wells contained DMSO (0.05%) or 500 nM chloroquine. 100 µl of parasite suspension (1% ring-infected erythrocytes at 4% haematocrit) was added to each well, and plates were incubated in a modular incubator chamber (Billups-Rothenberg, Inc.) with the gas mixture at 37°C for 50 hours. At the end of incubation, 100 µl culture medium was carefully removed from each well, and 100 µl lysis buffer (20 mM Tris-Cl, 5 mM EDTA, 0.008% saponin, 0.08% Triton X-100, pH 7.5) with SYBR Green 1 (at the manufacturer’s recommended dilution) was added to each well. The plate was incubated at 37°C for 30 min, and fluorescence was measured (Ex: 485 nm, Em: 530 nm, gain setting: 50) using an Infinite M200 multimode microplate reader (TECAN) as described previously [48]. The fluorescence of chloroquine-treated culture was subtracted from inhibitor-treated and DMSO-containing cultures to account for background fluorescence. Fluorescence values of inhibitor-treated cultures were normalized as percentage of DMSO-treated cultures, plotted against inhibitor concentrations, and analyzed using nonlinear regression analysis to determine IC_50_ concentrations for the inhibitors as described earlier [12].

For assessing the effect of inhibitor treatment on parasite morphology, ring stage parasites were cultured with DMSO (0.1%) or inhibitors (21.7 µM E64, 0.024 µM epoxomicin, 0.820 µM lactacystin, 0.133 µM MG132, and 220.6 µM pepstatin; concentrations are nearly 3 times the IC_50_) for 48 hours. Samples were collected at various time points, and thin smears were stained with Giemsa and observed under a 100 x lens using a bright field microscope.

To investigate the effect of inhibitors on different developmental stages, highly synchronized parasites (3% rings) were cultured for 60 hours in the presence of DMSO (0.25%) or inhibitors (21.7 µM E64, 0.024 µM epoxomicin, and 0.133 µM MG132; concentrations are nearly 3 times the IC_50_), which were added at different time points of the development. DMSO was added in the beginning (T0), and inhibitors were added in the beginning and at 12, 24 and 36 hour time points. Thin smears were prepared after 48 hours and 60 hours, stained with Giemsa, and observed using a bright field microscope under a 100 x objective. At least 200 parasites were observed to evaluate parasite morphologies and the number of different parasite stages.

To determine if MG132 irreversibly blocks parasite development, inhibitor washout experiments were performed for three development cycles (^≈^150 hours). Ring stage parasites (3% parasitemia) were cultured in the presence of DMSO (0.25%) or chloroquine (500 nM) or inhibitors (21.7 µM E64, 0.024 µM epoxomicin, 0.133 µM MG132; concentrations are nearly 3 times the IC_50_) for 50 hours in a 96-well tissue culture plate (250 µl culture volume, four wells for each treatment). The culture media was removed at the end of the incubation period; cells were washed twice with fresh culture medium, and then resuspended in 250 µl of fresh culture medium. 100 µl of this culture was used for measurement of parasite growth by the SYBR Green 1 assay, 50 µl was transferred to a well of another 96-well plate containing 200 µl culture medium with fresh erythrocytes (at 2% haemetocrit) for assessing parasite viability and growth, and the remaining culture was used for making smears for Giemsa-staining. For measuring parasite viability and growth, the plate was incubated in a modular incubator chamber (Billups-Rothenberg, Inc.) with the gas mixture at 37°C for 50 hours, and then each culture was processed for measurement of parasite growth by the SYBR Green 1 assay, assessing parasite viability and growth in the next development cycle, and making smears for Giemsa-staining as described above. The fluorescence values of chloroquine-treated, inhibitor-treated, and DMSO-containing cultures were compared to assess parasite growth. In parallel, Giemsa-stained smears were observed to assess parasite morphology and determine parasitemia. At least 1000 cells were counted to determine parasitemia, and 200 parasites were observed to assess morphology.

### Effect on hydrolysis of hemoglobin and ubiquitinated proteins

To test if MG132 targets hemoglobin hydrolysis and the UPS, a 100 ml culture of early trophozoite stage *P. falciparum* parasites (at 11% parasitemia) was divided into 4 equal parts; DMSO (0.1% final) was added to one part, and inhibitors (21.7 µM E64, 0.024 µM epoxomicin, and 0.133 µM MG132; all at concentrations nearly 3 times the IC_50_) were added to the remaining 3 parts. The cultures were grown for 10 hours, and parasites were isolated as described above. The parasite pellet from each culture was suspended in 150 µl reducing SDS-PAGE sample buffer, boiled for 10 min, and centrifuged at 27,216g for 15 min at room temperature, and the supernatants were then separated. For hemoglobin analysis, supernatants (corresponding to ^≈^2.5 x 10^7^ parasites) were run in 15% SDS-PAGE, and the gel was stained with coomassie blue. For analysis of ubiquitinated proteins, supernatants (corresponding to ^≈^5 x 10^7^ parasites) were run in 10% SDS-PAGE, and resolved proteins were transferred onto an Immobilon-P membrane (Millipore). The membrane was blocked (5% nonfat dry milk and 3% BSA in TBST (100 mM Tris-Cl, 0.5 M NaCl, 0.05% Tween-20, pH 7.5), incubated with mouse anti-ubiquitin antibodies (Santa Cruz Biotechnology, Cat No. sc-8017; 1/500 dilution in the blocking buffer), and then incubated with HRP-conjugated goat anti-mouse IgG (Invitrogen; diluted 1/10,000 dilution in blocking buffer). The signal was developed with the SuperSignal West Pico Chemiluminescent kit (Pierce) on X-ray film.

### MG132 cytotoxicity for human peripheral blood mononuclear cells (PBMCs)

10 ml human blood was collected aseptically in heparinized tubes, mixed with 10 ml complete medium (RPMI1640 with 10% FBS), layered over an equal volume of Histopaque-1077, and centrifuged at 400g for 20 minutes at room temperature. The interphase containing PBMCs was transferred to a fresh tube, mixed with 10 ml complete medium, and centrifuged at 350g for 10 minutes. The cells were washed twice with 10 ml complete medium. The washed cells were suspended in complete medium (5.5 x 10^5^ cells/ml) and grown for 20 hours at 37°C in a CO_2_ incubator. The cells were suspended in fresh culture medium, 100 µl cell suspension was added to the wells of a 96 well tissue culture plate (7 x 10^4^ cells/well), and the plate was incubated for 4 hours at 37°C in a CO_2_ incubator. 100 µl complete medium with 0.025% DMSO or varying concentrations of MG132 was added to the wells (three wells for DMSO and each concentration of MG132), and the plate was incubated in a 37°C CO_2_ incubator for 48 hours. MG132 cytotoxicity was determined following the quick protocol for MTT assay (Invitrogen). Briefly, 20 µl MTT (3-(4, 5-dimethylthiazolyl-2)-2, 5-diphenyltetrazolium bromide; 5 mg/ml in Hanks’ balanced salt solution (HBSS)) was added to each well, and the plate was incubated in a 37°C CO_2_ incubator for 3 hours to label cells. The plate was centrifuged at 894g for 5 minutes, the culture medium was removed, and the cells were washed with 150 µl of HBSS. The plate was centrifuged again at 894g for 5 minutes, and the supernatant was removed. The insoluble MTT product formazan in cells was solubilized with DMSO (150 µl/well), and absorbance was read at 540 nm for formazan and at 690 nm for plate background. The plate background was subtracted from the 540 nm reading, and the absorbance values of MG132-treated cultures were normalized as percentage of DMSO-treated cultures, plotted against MG132 concentrations, and analyzed using the Four Parameter Logistic Function dose response curve (Sigma Plot) to determine the 50% cytotoxic concentration.

### Effect of MG132 on falcipain-2 knockout parasites

To determine susceptibility of FP2 knockout (FP2KO) parasites to MG132, the knockout and parental wild type parasites were cultured in the absence (control) or presence of various concentrations of MG132 or chloroquine in RPMI 1640 containing 10% human serum at 37°C for 50 hours. Parasitemias in control and inhibitor-treated cultures were determined by flow cytometry and compared to determine IC_50_ values of inhibitors as described earlier [7,12].

### Effect of inhibitor combinations on parasite growth

To determine the effect of inhibitor combinations on parasite growth, 300 x stocks of combinations (E64-MG132, epoxomicin-lactacystin, E64-epoxomicin, and E64-lactacystin in the ratio of 1:5, 1:3, 1:1, 3:1, and 5:1 of their IC_50s_; E64-pepstatin, MG132-pepstatin, and epoxomicin-pepstatin in the ratio of 2:1 and 1:1 of their IC_50s_) were serially diluted 2-fold in 100 µl culture medium across rows of a 96 well tissue culture plate. 100 µl of parasite suspension (1% ring-infected erythrocytes at 4% haematocrit) was added to each well, cultures were grown for 50 hours, and the 50% inhibitory concentration of each inhibitor in the combination was determined as described above. The 50% inhibitory concentration of each inhibitor in the combination was used to determine the fractional inhibitory concentration (FIC) as described previously (FIC = concentration of a compound that caused 50% inhibition in the combination/concentration of the compound required for 50% inhibition when used alone) [49]. The FICs obtained at different combination ratios of compound 1 and compound 2 were plotted using linear regression analysis (GraphPad Prism) to construct an isobologram, and the two axes of the isobologram were connected at points corresponding to FIC 1.0. The sum of FICs (ΣFIC: FIC of compound 1 + FIC of compound 2 in the combination) at all combinations was used to determine mean ΣFIC, which was used to determine the nature of interactions (ΣFIC 0.5-1.0, <0.5, and >1.5 are indicative of additive, synergistic and antagonistic interactions, respectively).

### Inhibition assay with parasite extracts

A synchronized *P. falciparum* culture with trophozoite/schizont stage parasites (10% parasitemia) was processed for isolation of parasites as described above. The parasite pellet was suspended in 600 µl lysis buffer (10 mM Tris, pH 7.0, 1 mM EDTA, 1 mM PMSF, 1 mM DTT, 1 mM ATP, and 10 µM pepstatin), subjected to 2 cycle of freeze-thaw (in liquid N_2_) and sonication for 3 min (pulses of 9 seconds on/off and 20% amplitude at 4°C; Sonics). The lysate was centrifuged at 13,000g for 15 min at 4°C, the supernatant was transferred to a fresh tube, its protein content was estimated using Bio-Rad protein assay reagent (Bio-Rad), and it was used for inhibition assays. Identical aliquots of the extract (corresponding to 16 µg protein) were added to 100 µl cysteine protease assay buffer (100 mM sodium acetate, pH 5.5, 10 mM DTT, 1 mM EDTA, 1 mM PMSF, 10 µM pepstatin) or proteasome assay buffer (50 mM Tris, 1 mM ATP, 5 mM MgCl_2_, 40 mM KCl, 1 mM DTT, 1 mM PMSF, 10 µM pepstatin, pH 7.5), which contained DMSO (1.25% final) or individual inhibitors (10 µM E64, 1 µM epoxomicin, or 1 µM MG132) or combinations of inhibitors (10 µM E64 + 1 µM epoxomicin, 10 µM E64 + 1 µM MG132, or 1 µM epoxomicin + 1 µM MG132). The reactions were incubated for 10 min at 37°C, substrates (Z-LR-AMC for cysteine protease activity, Suc-LLVY-AMC for chymotrypsin-like proteasome activity, Ac-nLPnLD-AMC for caspase-like proteasome activity, and Ac-RLR-AMC for trypsin-like proteasome activity) were added to the reactions, and protease activities were determined by monitoring substrate hydrolysis for 30 min at 37°C using an Infinite M200 (TECAN) or SpectraMax M5 (Molecular Devices) microplate reader (excitation 355 nm; emission 460 nm). Enzyme activities with Z-LR-AMC and Ac-RLR-AMC were expressed as relative fluorescence units/min (RFU/min), and those with Suc-LLVY-AMC and Ac-nLPnLD-AMC were expressed as total increase in RFU over 30 min. Enzyme activities of inhibitor-containing reactions were compared with those of DMSO-containing reactions, expressed as percent inhibition, and plotted against the inhibitors.

### Assays with recombinant falcipains

For production of recombinant FP2 and FP3, portions of the FP2 and FP3 genes corresponding to the carboxy-terminus regions of the prodomains (FP2, 36 amino acid residues; FP3, 35 amino acid residues) and the entire mature protease domains were PCR amplified from 3D7 gDNA and expressed in bacteria using pRSETA (for FP2) and pQE-30 (for FP3) expression plasmids. Recombinant proteins were purified from isopropyl-1-thio-β-D-galactopyranoside (IPTG) induced bacteria by nickel-nitrilotriacetic acid (Ni-NTA) chromatography under denaturing conditions as described earlier [15,50]. The purified FP2 and FP3 were refolded into active forms as described earlier [15,50], with a modification that involved dialysis of denatured proteins against refolding buffers, and used for inhibition assays. Concentrations of active FP2 and FP3 were determined by active site titration using E64. Briefly, enzymes were incubated with DMSO (1% final) or varying concentrations of E64 in sodium acetate buffer (100 mM sodium acetate, 10 mM DTT, pH 5.5) for 30 min at room temperature, Z-LR-AMC was added (25 µM final) to the reactions, and enzyme activity was determined by monitoring the release of AMC upon substrate hydrolysis over 30 min at 37°C as described above. Enzyme activities as RFU/min for DMSO and inhibitor-containing reactions were plotted against inhibitor concentrations, and analyzed using the ORIGIN program (OriginLab) to determine enzyme concentrations.

To determine if FP2 and FP3 are inhibited by proteasome inhibitors, FP2 (5 nM) or FP3 (10 nM) was incubated with DMSO (1% final) or with inhibitors (MG132, epoxomicin, lactacystin, E64, and pepstatin; each at 10 µM) in sodium acetate assay buffer for 30 min at room temperature. Z-LR-AMC was added to the reaction (25 µM final), and enzyme activity was measured by monitoring substrate hydrolysis for 30 min at 37°C as described above. Enzyme activities of inhibitor-containing reactions were compared with those of DMSO-containing reactions, expressed as percent inhibition, and plotted against the inhibitors.

To determine association rate constants of inhibitors for FP2 and FP3, enzymes (1 nM FP2 or 2 nM FP3) were added to 200 µl reactions (100 mM sodium acetate, pH 5.5, 10 mM DTT, 25 µM Z-LR-AMC) with DMSO (1% final) or varying concentrations of E64 (at concentration >10 times the enzyme concentration) or MG132 (2-10 nM), and enzyme activity was measured by monitoring the release of AMC upon substrate hydrolysis for 30 min at 37°C as described above. The activity progress curves (fluorescence vs time) without and with inhibitors were analyzed by non-linear regression analysis (GraphPad software) using the pseudo-first-order equation.

*y* = *A*[1 − exp(−*k*_obs_ *t*)] + *B*; where *y* is the fluorescence intensity at time *t*, *A* is the amplitude of the reaction, and *B* is the offset. *k*_obs_ vs inhibitor concentration [I] was linear. The association rate constant *k*_ass_ was determined by linear regression using *k*_ass_ = (*k*_obs_/[I])(1 + [S]/*K*_m_); where [S] is the substrate concentration.

## Results and Discussion

### MG132 inhibits *P. falciparum* development, hemoglobin degradation and the UPS

**Figure 1:**
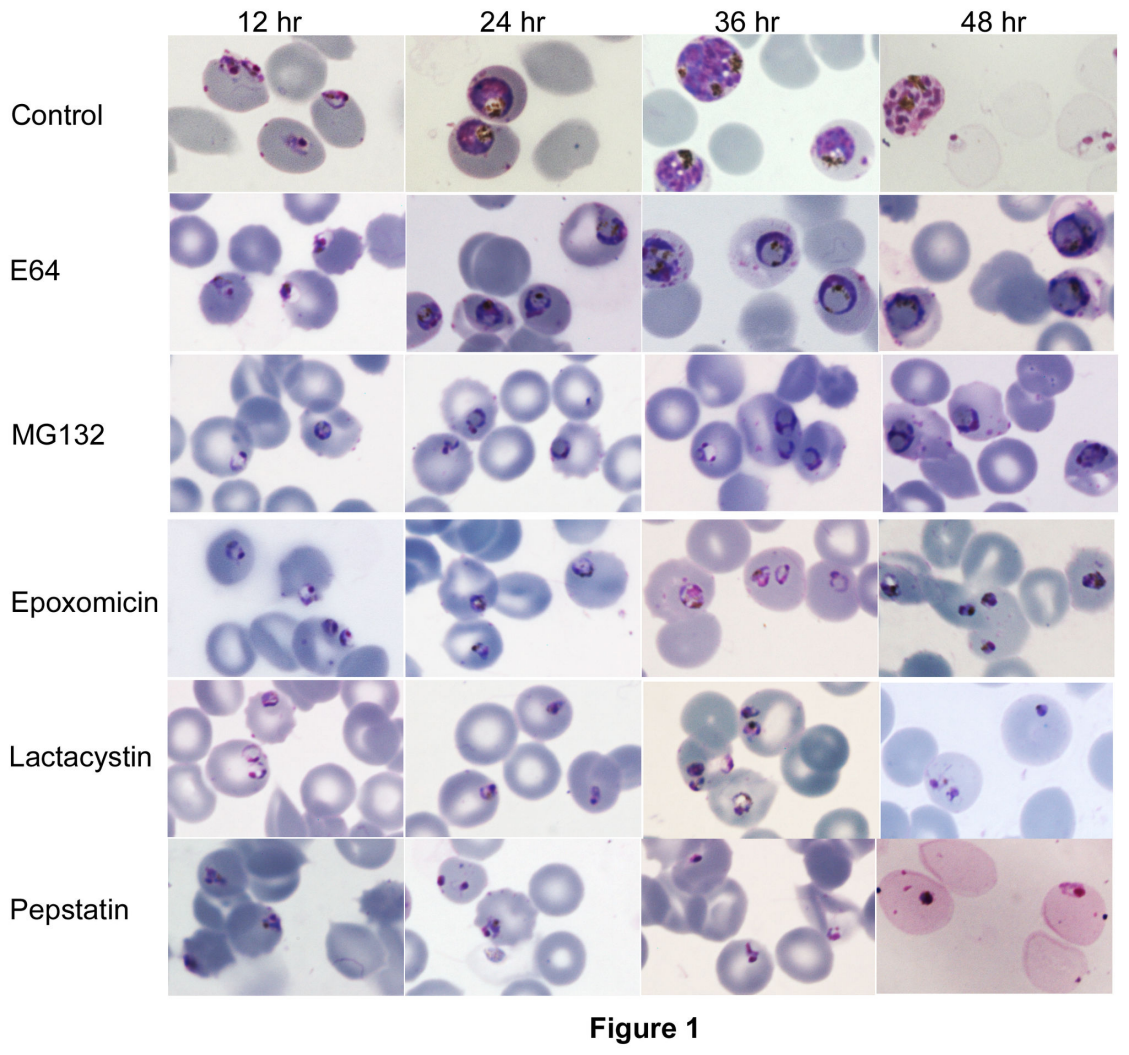
Morphologies of inhibitor-treated parasites. Parasites were cultured without (control) or with indicated inhibitors (21.7 µM E64, 0.024 µM epoxomicin, 0.812 µM lactacystin, 0.133 µM MG132, and 220.6 µM pepstatin; concentrations are nearly 3 times the IC_50_) for 48 hours, and the morphologies of parasites on Giemsa-stained smears were evaluated at indicated time points. Note that both E64- and MG132-treated parasites have enlarged food vacuoles occupying almost the entire parasite, whereas parasites treated with epoxomicin, lactacystin, and pepstatin are pyknotic.

To determine if MG132 exerts antimalarial activity by inhibition of hemoglobin degradation and/or by inhibiting the UPS, we assessed the effects of inhibitors of falcipain cysteine proteases (E64), the UPS (epoxomicin, lactacystin, and MG132), and plasmepsin aspartic proteases (pepstatin) on *P. falciparum* erythrocytic development. All these inhibitors blocked parasite development (Table 1), but the inhibited parasites showed different morphologies. As has been previously described, E64 caused enlargement of food vacuoles (Figure 1 [9]), which is indicative of accumulation of undegraded hemoglobin due to inhibition of falcipains. MG132, but not epoxomicin or lactacystin, also caused enlargement of the food vacuole, suggesting that MG132, like E64, inhibited hemoglobin degradation. However, MG132-treated parasites were much smaller than E64-treated parasites. Pepstatin, an inhibitor of the food vacuole-resident plasmepsins, resulted in pyknotic parasites without swollen food vacuoles. This result is consistent with previous studies demonstrating normal hemoglobin catabolism in plasmepsin knockout parasites [51,52].

**Table 1:** Inhibition of intraerythrocytic parasite development.

**Inhibitors**	**IC_50_** ^a^ (µM)
E64	7.6 ± 1.22
Epoxomicin	0.0077 ± 0.00027
Lactacystin	0.276 ± 0.038
MG132^b^	0.0476 ± 0.0058
Pepstatin	74.3 ± 8.8

^a^ Mean IC_50_ with standard deviation from three independent experiments, each in duplicate.

^b^ The IC_50_ for PBMCs was 10.8±0.04 µM.

The 50% cytotoxic concentration of MG132 for PBMCs was 10.8 µM (Table 1), which is remarkably higher than its IC_50_ for inhibition of parasite growth (0.0476 µM), and indicates its selective toxicity for malaria parasites. The previously reported 50% cytotoxic concentration of MG132 for PMBCs was somewhat higher (20 µM), but was based on a shorter (24 hour) incubation with the inhibitor [43].

**Figure 2:**
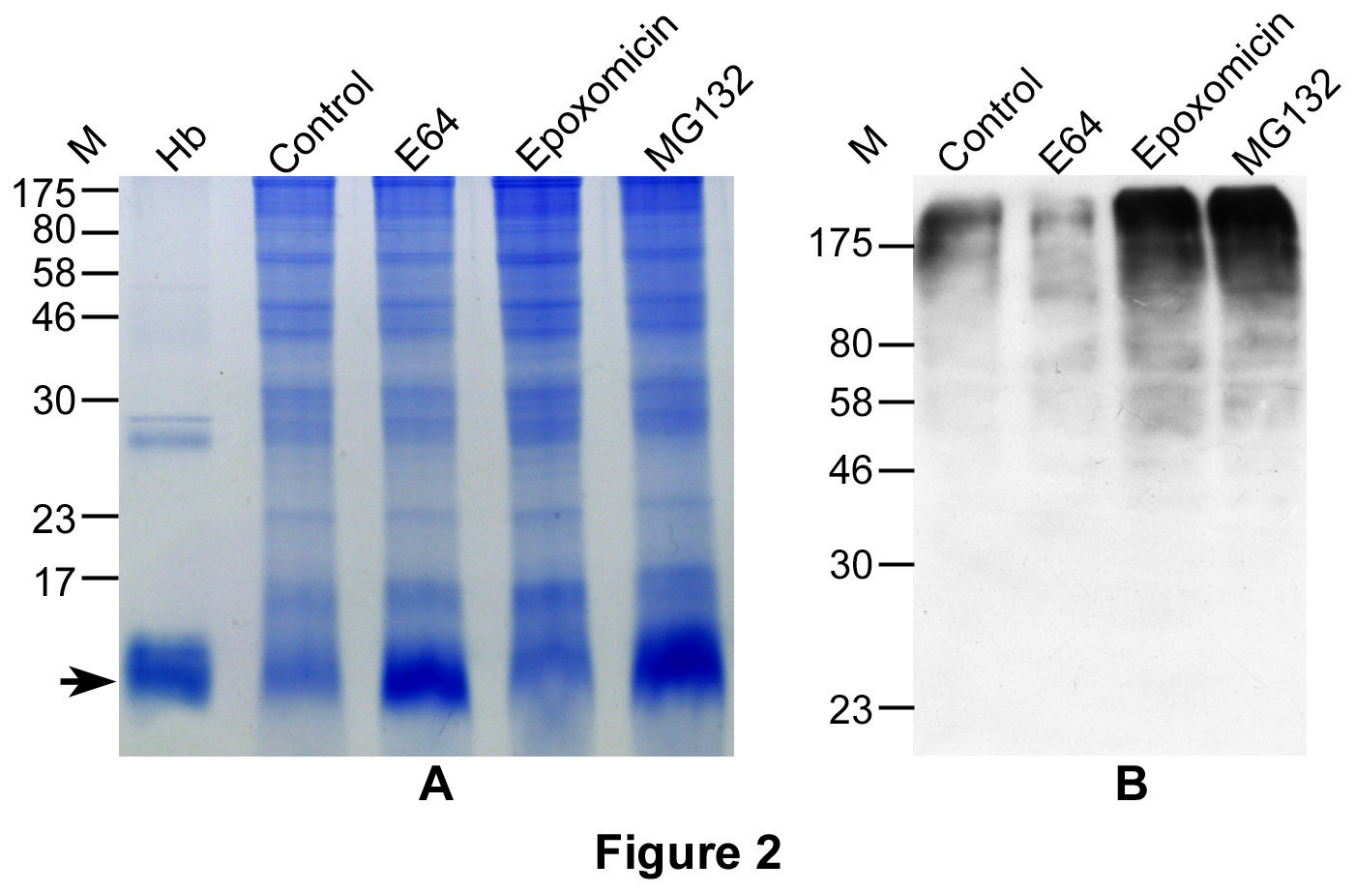
Accumulation of undegraded hemoglobin and ubiquitinated proteins in inhibitor-treated parasites. Cultures containing equal number of early trophozoite stage parasites were grown in the presence of 0.1% DMSO (control) or indicated inhibitors (21.7 µM E64, 0.024 µM epoxomicin, and 0.133 µM MG132; all at concentrations nearly 3 times the IC_50_) for 10 hours. Parasites were lysed and equal amounts of supernatants were used to assess accumulation of undegraded hemoglobin (A) and ubiquitinated proteins (B) as described in the Materials and Methods section. **A**. Coomassie-stained SDS-PAGE gel, showing significantly more amount of undegraded hemoglobin (marked with arrow) in parasites treated with E64 and MG132 than control and epoxomicin-treated parasites. **B**. Western blot using anti-ubiquitin antibodies showed markedly more intense high molecular weight signal in the MG132 and epoxomicin samples than in control and E64 samples, which is indicative of accumulation of ubiquitinated proteins in the MG132 and epoxomicin samples. The experiment was repeated twice, samples from each experiment were analyzed three times for A and two times for B, and the results were reproducible. M, molecular weight in kD; Hb, hemoglobin.

To determine if MG132-treated parasites had accumulated undegraded hemoglobin, total lysates of control and inhibitor-treated parasites were analyzed by SDS-PAGE. Both E64- and MG132-treated parasites, but not controls and those treated with epoxomicin, showed significant accumulation of undegraded hemoglobin (Figure 2A), confirming inhibition of hemoglobin degradation by MG132. Immunoblots with anti-ubiquitin antibodies showed a high molecular weight signal, which was markedly more intense in lanes containing MG132- and epoxomicin-treated parasites than lanes with control and E64-treated parasites (Figure 2B), indicating accumulation of ubiquitinated proteins upon inhibition of the UPS. Thus, unlike the other inhibitors tested, MG132 appears to inhibit both UPS and hemoglobin degradation, and both of these activities likely contribute to its action against asexual *P. falciparum* erythrocytic development.

### MG132 is effective against all asexual erythrocytic stages and irreversibly blocks parasite development

To determine if MG132 is effective against all erythrocytic stages, parasites at different stages of development (ring, T0 and T12; early trophozoite, T24; late trophozoite, T36) were treated with inhibitors (E64, epoxomicin, and MG132), and the number of newly formed rings was counted at the end of the development cycle (48 hours) and 12 hours after the end of the cycle (60 hour). The control parasites developed normally and formed new rings upon completion of the development cycle (Table 2). Treatment with MG132 and epoxomicin at ring and early trophozoite stages completely blocked the development, and that with E64 resulted in a very small number of new rings (Table 2). Treatment at the late trophozoite stage also had significant inhibitory effect, as only 3-11% of the total parasite population was of new rings (Table 2). The number of rings in the control increased from 40% at 48 hour time point to 80% at 60 hour time point; the inhibitor-treated parasites had 0-11% rings at 48 hour time point, and this number almost remained the same (0-12%) at 60 hour time point regardless of the stage when inhibitors were added, which suggests that inhibitors blocked maturation of parasites. Both E64 and MG132, but not epoxomicin, caused food vacuole enlargement in the majority (88%) of parasites regardless of the treatment time point. Thus, MG132 inhibits development of all asexual erythrocytic stages.

**Table 2:** Stage-specific effects of inhibitors.

**Post synchronization stage at which treatment started**	**% Rings (R) at 48 hours and 60 hours post-synchronization in cultures containing DMSO (control) or inhibitors**			
	**^a^Control**	**E64**	**MG132**	**Epoxomicin**
	**48/60 hours**	**48/60 hours**	**48/60 hours**	**48/60 hours**
**0 Hour**	41 (±15)/80 (±12)	5 (±3)/5 (±0)	0/0	0/0
**12 Hour**	-	6 (±6)/5 (±2)	0/0	0/0
**24 Hour**	-	6 (±6)/6 (±1)	0/0	0/0
**36 Hour**	-	11 (±3)/12 (±0)	3 (±3)/10 (±0)	6 (±6)/0

Ring stage parasites were treated with DMSO (control) or inhibitors (21.7 µM E64, 0.024 µM epoxomicin, and 0.133 µM MG132; all at concentrations nearly 3 times the IC_50_) at different stages of development (ring, T0 and T12; early trophozoite, T24; late trophozoite, T36), and the number of rings was counted at the end (48 hours) and 12 hours after the end (60 hours) of the development cycle. At least 200 parasite-infected cells were observed, and the table shows the percentage of rings for control and the inhibitor-treated cultures. The data are mean with standard deviation from three (for 48 hour) and two independent experiments (60 hour) done in duplicates.

^a^ DMSO was added at the start of the experiment (T0) only.

**Figure 3:**
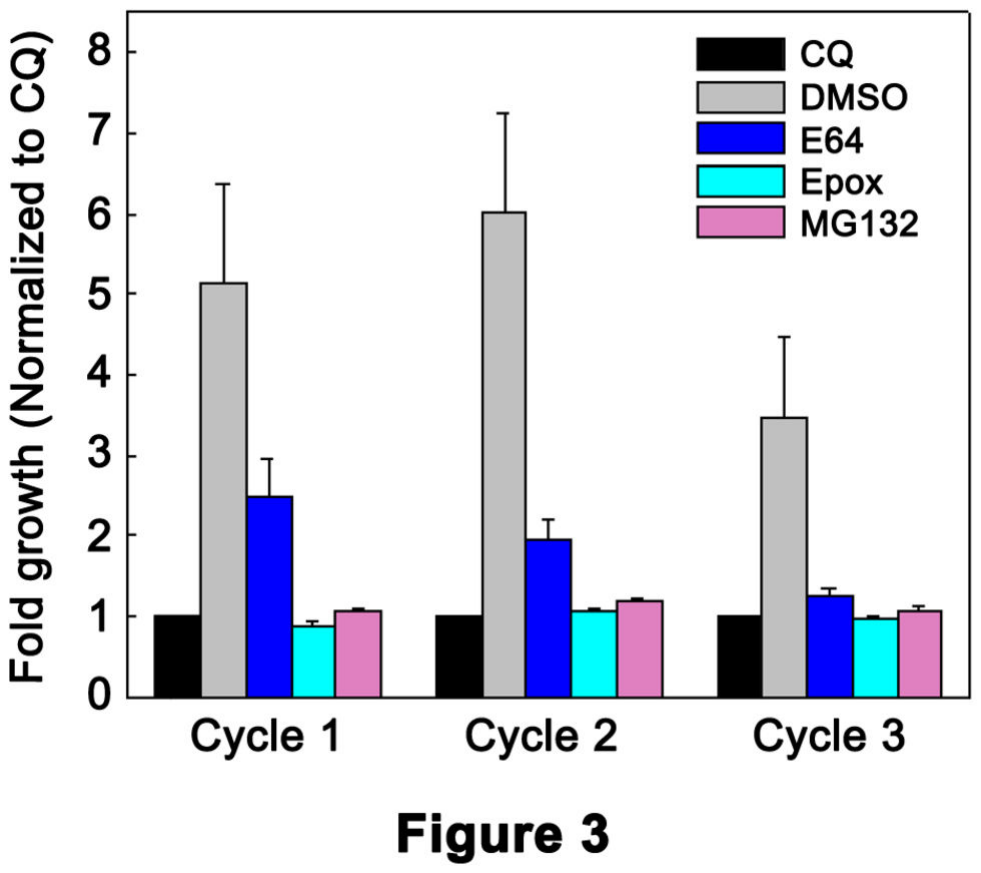
Effect of inhibitor washout on parasite growth. Ring stage parasites were cultured in the presence of DMSO (control) or chloroquine or indicated inhibitors (21.7 µM E64, 0.024 µM epoxomicin, 0.133 µM MG132; concentrations are nearly 3 times the IC_50_) for one cycle, and then without any treatment for next two cycles as described in the Materials and Methods section. Parasite growth was measured at the end of each cycle using the SYBR Green-1 dye. The results with standard deviation error bars from three independent experiments, each in triplicate, are shown as arbitrary fluorescence units of the dye. Similar fluorescence values of parasites treated with MG132, epoxomicin, and chloroquine indicate that MG132 and epoxomicin, like chloroquine, irreversible block parasite development.

To test if the inhibition of parasite development by MG132 is permanent we performed inhibitor washout experiments in which parasites were cultured for one cycle in the presence of DMSO (control) or chloroquine (an irreversible inhibitor) or inhibitors (MG132, E64, and epoxomicin), and then without any treatment for two cycles. In SYBR Green-based assay, which is a measure of overall parasite growth, control parasites showed almost 3.5-6 times higher fluorescence than chloroquine-treated parasites in all three cycles (Figure 3), which indicated robust growth in the control culture. The E64-treated parasites showed similar to 2.5 times higher fluorescence than chloroquine-treated parasites, particularly in cycle 1, which suggested partial growth during treatment and recovery of parasites after E64 was washed out. The fluorescence values of MG132- and epoxomicin-treated parasites were similar to that of chloroquine-treated parasites at the end of all three cycles, which suggested that both MG132 and epoxomicin irreversibly blocked parasite development in cycle 1. Giemsa-stained smears of the control culture showed 3.5-6 fold increase in parasitemia (10 to 18% parasitemia) over the starting parasitemia in all three cycles, which is consistent with the results of SYBR Green assay. Parasitemia did not increase in cultures containing the inhibitors and chloroquine at the end of cycle 1. Dark-stained dots (cycle 2) or no parasites (cycle 3) were observed in MG132- and epoxomicin-treated cultures after the inhibitors had been washed out, indicating that MG132 and epoxomicin, like chloroquine, irreversibly block parasite development. On the other hand, washing out E64 led to partial recovery of a fraction of parasites in subsequent cycles (^≈^1% parasitemia). Thus, both SYBR Green assay and the assessment of Giemsa-stained parasite smears indicate that MG132 irreversibly blocks parasite development.

### MG132 inhibits the UPS and cysteine protease activities in parasites

To further test the predicted actions of MG132, we determined whether cysteine protease and proteasome activities of a total soluble parasite extract are inhibited by MG132. FP2 and FP3 contribute almost exclusively to the acidic cysteine protease activity of *P. falciparum* erythrocytic stages; this is routinely measured by monitoring hydrolysis of Z-LR-AMC and is blocked by the cysteine protease inhibitor E64. Malaria parasites appear to contain a typical 26S proteasome, but the proteasome-associated activities have not been characterized. A typical 26S proteasome possesses three types of activities [33–35]: peptidyl-glutamyl peptide hydrolyzing (PGPH) activity that cleaves after acidic residues; trypsin-like activity that cleaves after basic residues; and chymotrypsin-like activity that cleaves after large hydrophobic residues. These three activities are routinely measured using fluorogenic peptide substrates (Z-nLPnLD-AMC for PGPH, Ac-RLR-AMC for trypsin, and Suc-LLVY-AMC for chymotrypsin) [53]. The chymotrypsin activity is inhibited by the majority of proteasome inhibitors, including MG132 and epoxomicin [36–39]. Control inhibitors of other proteases (EDTA, metalloproteases; PMSF, serine proteases; and pepstatin, aspartic proteases) were included in all reactions.

**Figure 4:**
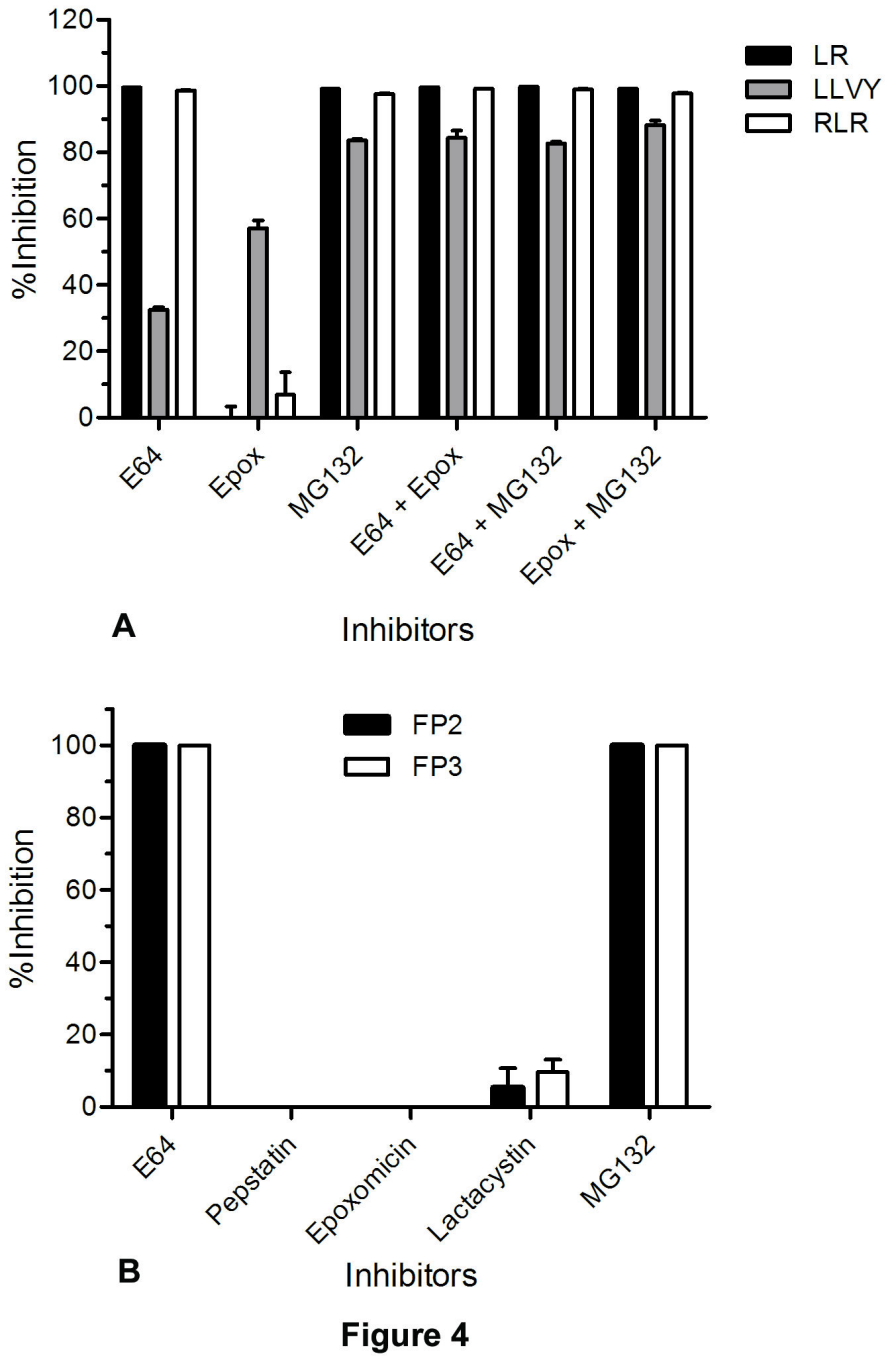
Inhibition of cysteine protease and proteasome activities by MG132. **A**. Inhibition of total soluble extracts of *P. falciparum*. Extracts of trophozoite/schizont stage parasites were prepared by freeze-thaw lysis and ultrasonic treatment of cells. Identical aliquots were treated with 1.25% DMSO (control), 10 µM E64, 1 µM epoxomicin (Epox), 1 µM MG132, or the indicated inhibitor combinations (10 µM E64 + 1 µM epoxomicin, 10 µM E64 + 1 µM MG132, or 1 µM epoxomicin + 1 µM MG132) for 10 min at 37°C, and protease activities were determined by monitoring hydrolysis of fluorogenic peptide substrates for 30 min at 37°C as described in Materials and Methods. The substrates used were Z-LR-AMC (LR) for cysteine protease activity, Suc-LLVY-AMC (LLVY) for chymotrypsin-like proteasome activity, and Ac-RLR-AMC (RLR) for trypsin-like proteasome activity. Activities of inhibitor-containing reactions were compared with those of controls and expressed as percent inhibition. The results shown are means of two independent experiments, each performed in duplicate. **B**. Inhibition of recombinant falcipains. Recombinant FP2 (5 nM) or FP3 (10 nM) was incubated with 1% DMSO (control) or indicated inhibitors (all 10 µM) in sodium acetate assay buffer for 30 min at room temperature. Z-LR-AMC (25 µM) was added to the reaction, and enzyme activity was measured by monitoring substrate hydrolysis for 30 min at 37°C. Enzyme activities of inhibitor-containing reactions were compared with those of controls and expressed as the percent inhibition. The results shown are means of three independent experiments, each performed in triplicate.

E64 and MG132 completely inhibited Z-LR-AMC hydrolyzing activity of the extract, suggesting that MG132, like E64, inhibits falcipains (Figure 4A). Epoxomicin alone did not inhibit Z-LR-AMC hydrolyzing activity of the extract, but it completely inhibited the same activity when combined with E64 or MG132, suggesting that it does not inhibit falcipains.

The hydrolysis of the chymotrypsin substrate Suc-LLVY-AMC was inhibited by E64, MG132, and epoxomicin to varying degrees (Figure 4A). As E64 and epoxomicin specifically target falcipains and UPS, respectively, it seems that both falcipains and the UPS contribute to the total Suc-LLVY-AMC hydrolysing activity. MG132 alone was as effective (83.6%) as E64-epoxomicin combination (84.3%) in inhibiting the Z-LR-AMC hydrolyzing activity of the extract, suggesting that MG132 has both cysteine protease and proteasome inhibitory activities. This dual-inhibitory property of MG132 also explains comparable inhibition of Suc-LLVY-AMC hydrolysis by MG132 alone and together with E64 (83%) or epoxomicin (88%).

The parasite extract showed negligible hydrolysis of the PGPH substrate Z-nLPnLD-AMC (1.75 RFU/min), which may be due to different substrate preference of the Plasmodium proteasome. All three inhibitors marginally inhibited Z-nLPnLD-AMC hydrolyzing activity, and the inhibition levels were not clearly distinguishable, hence the data is excluded from Figure 4A.

Hydrolysis of the trypsin substrate Ac-RLR-AMC was almost completely inhibited by E64 and MG132, and only slightly inhibited by epoxomicin, suggesting that falcipains primarily contributed to the hydrolysis of Ac-RLR-AMC. As in the case of Z-LR-AMC hydrolysis, combinations of E64, MG132, and epoxomicin were as effective as E64 or MG132 alone. Complete inhibition of Ac-RLR-AMC hydrolysing activity by MG132 alone, as by the specific cysteine protease inhibitor, further supports the falcipain-inhibitory property of MG132. The hydrolysis of Ac-RLR-AMC by falcipains was not surprising, as these and other papain-family enzymes readily hydrolyze substrates with Arg or Lys at the P1 position [18]; falcipains prefer Leu at the P2 position and can also degrade peptide substrates containing P3 Arg [19].

Taken together, the demonstration of inhibition of both cysteine protease and UPS activities by MG132 and the accumulation of undegraded hemoglobin and ubiquitinated proteins in parasites after treatment with this compound are all consistent with the conclusion that MG132 blocks parasite development by inhibiting hemoglobin degradation and the UPS.

### MG132 is a potent falcipain inhibitor

As MG132 caused accumulation of undegraded hemoglobin in parasites and inhibited cysteine protease activity of the parasite extract, we assessed it for inhibition of recombinant FP2 and FP3. MG132 and E64 inhibited hydrolysis of Z-LR-AMC by both enzymes (Figure 4B), confirming that MG132 is a falcipain inhibitor. Identical concentrations of epoxomicin and pepstatin did not inhibit, and of lactacystin marginally inhibited the enzymes. A kinetic analysis revealed that, compared to E64, MG132 is 18 and 100 times more potent in the inhibition of FP2 and FP3, respectively (Table 3). Furthermore, MG132 was twice as effective against FP3 as FP2, which is of importance for drug development efforts, because only FP3 seems to be essential for erythrocytic development of *P. falciparum* [7,12].

**Table 3:** Association rate constants (M^-1^. S^- 1^) for recombinant falcipains^a^.

**Inhibitors**	**FP2**	**FP3**
E64	10,260 ± 714	4,090 ± 277
MG132	188,570 ± 24,840	412,460 ± 35,080

^a^ Means ± standard deviations for at least three independent experiments, each in duplicate.

Previous studies have demonstrated that loss of FP2 in FP2 knockout parasites renders them almost 2.5 times more sensitive than wild type parasites to E64 [7,12]. We evaluated FP2 knockout parasites for sensitivity to MG132. The FP2 knockout parasite was as susceptible as the wild type parasite to the control drug chloroquine (IC_50_: FP2KO, 5.3±1.5 nM; wild type, 4.5±0.3 nM), but almost twice as susceptible to MG132 (IC_50_: FP2KO, 15.5±1.4 nM; wild type, 26.6±4.1 nM), which further supports inhibition of falcipains by MG132.

### Joint inhibition of falcipains and UPS has an additive antiparasitic effect

**Figure 5:**
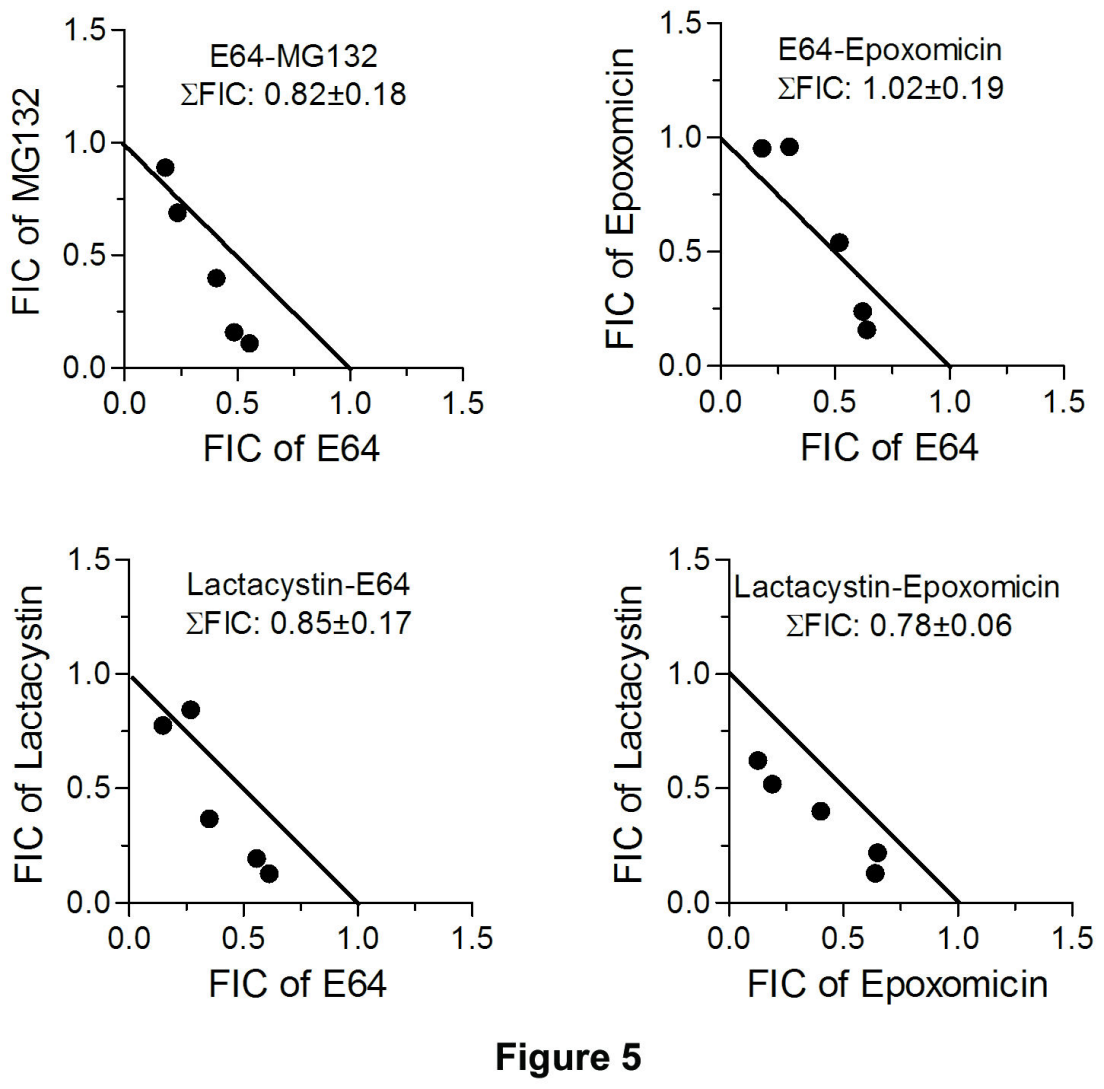
Antimalarial effects of combinations of proteasome and falcipain inhibitors. Parasites were cultured in the presence of varied combinations of the indicated inhibitor, and the 50% inhibitory concentration of each combination was determined to calculate fractional inhibitory concentrations (FIC). FICs from three independent experiments, each carried out in duplicate were used to construct isobolograms as described in Materials and Methods section. Mean FICs (ΣFIC) 0.5-1.0, <0.5, and >1.5 are indicative of additive, synergistic and antagonistic interactions, respectively. **A**. The isobolograms indicate additive interactions for the indicated combinations.

**Figure 6:**
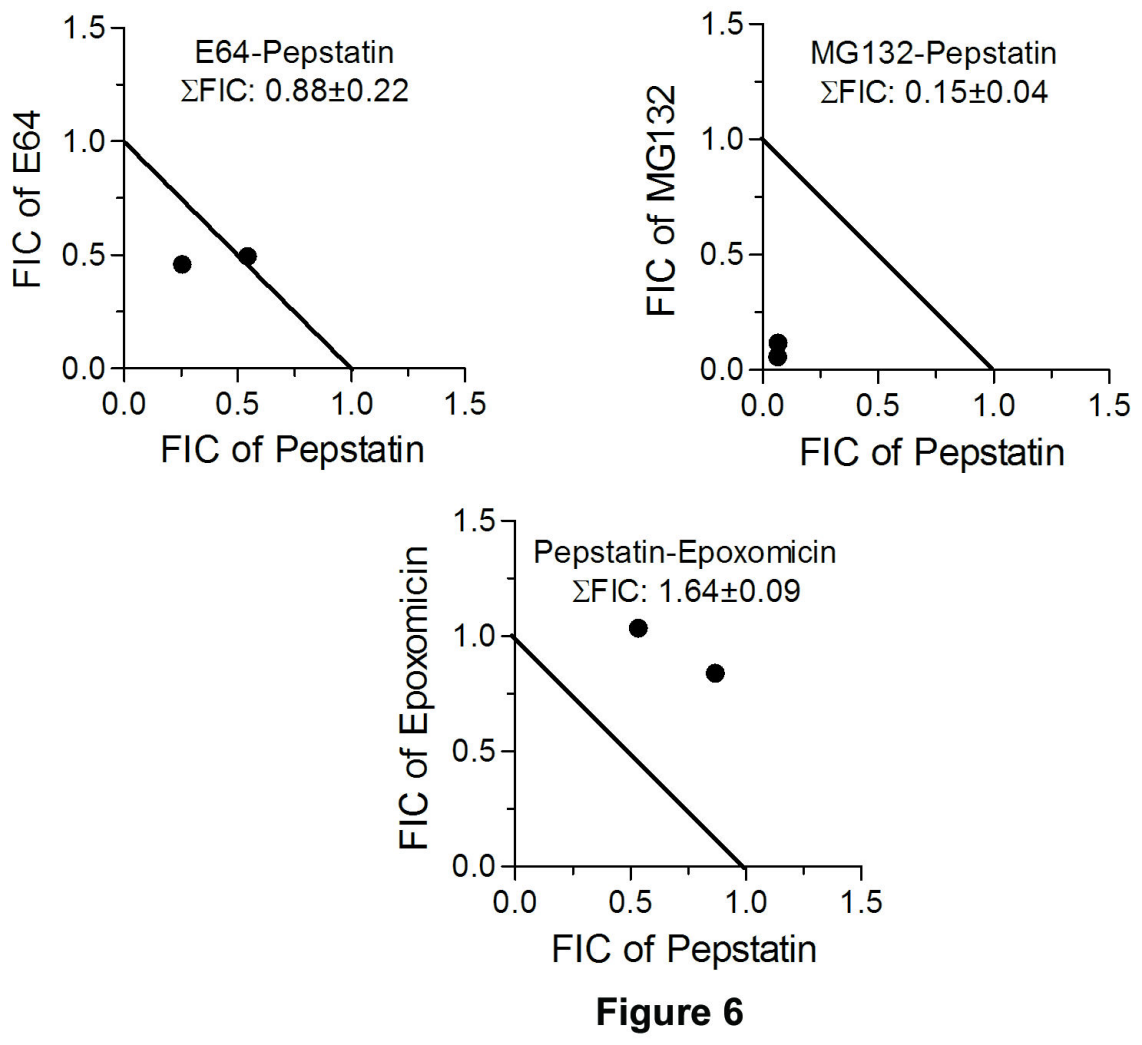
See figure 5 for legend. The isobolograms indicate additive, synergistic, and antagonistic antiparasitic effects of E64-pepstatin, MG132-pepstatin, and epoxomycin-pepstatin combinations, respectively.

A comparison of the IC_50s_ of MG132 and E64 for parasite inhibition (Table 1) indicated that MG132 is 160 times more potent than E64, which could be explained by superior potency for fa1cipains, improved cell permeability/intracellular accumulation, and simultaneous inhibition of both falcipains and UPS by MG132. Malaria parasite genomes encode 115–137 putative proteases of all five classes (aspartic, cysteine, metallo, serine, and threonine), and MG132 may target other proteases in addition to falcipains and UPS [54,55]. To further explore effects on the two targets, we evaluated antiparasitic effects of inhibitors of the proteasome (epoxomicin, lactacystin, and MG132), falcipains (E64), and plasmepsins (pepstatin). Combinations of proteasome-specific inhibitors (epoxomicin-lactacystin) showed additive effects, which was expected as both the inhibitors target the same enzyme (Figure 5). Combinations of E64 with epoxomicin, lactacystin or MG132 also had additive effects (Figure 5). Effects were more remarkable with pepstatin, as combination with MG132 showed synergy, with E64 showed a trend towards synergy, and with epoxomicin showed antagonism (Figure 6). Synergistic antiparasitic effects of MG132-pepstatin and E64-pepstatin agree with proposed joint roles of falcipains and plasmepsins in hemoglobin degradation, and were consistent with a previous result showing synergism of E64 and pepstatin [56]. The antagonistic effect of pepstatin-epoxomicin is unexplained. One explanation could be the accumulation of plasmepsins, which are major food vacuole aspartic proteases, in the presence of epoxomicin, as plasmepsin II has been shown to be a target for ubiquitin-proteasome-mediated degradation [57]. The accumulation of plasmepsins would decrease susceptibility of parasites to pepstatin, potentially mediating the antagonistic effect of the pepstatin-epoxomicin combination. The additive to slightly synergistic anti-parasitic effect of the UPS and falcipain inhibitors suggests therapeutic benefits of either combined chemotherapy based on the UPS and falcipain targets or a single agent, such as MG132, that inhibits both targets.

MG132 has also been shown to inhibit calpains, with IC_50_ values of 1.2 µM for purified and >100 µM for intracellular calpains [45]. However, as MG132 inhibits parasite growth at much lower concentrations (0.0476 µM), it is unlikely that inhibition of parasite and/or host calpains by MG132 contributes to its antimalarial effects.

Inhibition of the UPS is being actively pursued as a strategy to treat cancer; bortezomib is a proteasome inhibitor approved for treating multiple myeloma. Bortezomib has also been shown to kill *P. falciparum* intraerythrocytic stages with an IC_50_ concentration 2-4 times lower than levels achieved at recommended doses in human plasma [1], supporting consideration of proteasome inhibitors as antimalarials. MG132 is an inexpensive and potent inhibitor of the UPS, and for these reasons it has been widely studied as a proteasome inhibitor in a number of cell types, including several protozoan parasites. The reported 50% cytotoxic concentrations of MG132 for mammalian cell lines are in the range of 2.5-21 µM depending on the cell type and treatment duration [40–43]. In our studies MG132 was over 200 times more potent for inhibition of development of malaria parasites (IC_50_ 0.0476 µM) than for human PBMCs (10.8 µM), indicating that it is selectively toxic to the parasite. This selective toxicity could be due to the simultaneous inhibition of the two vital proteolytic systems of the parasite, the UPS and the falcipains, establishing it as a dual-target inhibitor of malaria parasites. MG132 may thus be considered a lead compound to design potent antimalarials acting against two parasite proteolytic systems.
